# Jacobian Maps Reveal Under-reported Brain Regions Sensitive to Extreme Binge Ethanol Intoxication in the Rat

**DOI:** 10.3389/fnana.2018.00108

**Published:** 2018-12-11

**Authors:** Qingyu Zhao, Michael Fritz, Adolf Pfefferbaum, Edith V. Sullivan, Kilian M. Pohl, Natalie M. Zahr

**Affiliations:** ^1^Department of Psychiatry and Behavioral Sciences, Stanford University School of Medicine, Stanford, CA, United States; ^2^Neuroscience Program, SRI International, Menlo Park, CA, United States

**Keywords:** alcohol, addiction, rodent, colliculus, thalamus

## Abstract

Individuals aged 12–20 years drink 11% of all alcohol consumed in the United States with more than 90% consumed in the form of binge drinking. Early onset alcohol use is a strong predictor of future alcohol dependence. The study of the effects of excessive alcohol use on the human brain is hampered by limited information regarding the quantity and frequency of exposure to alcohol. Animal models can control for age at alcohol exposure onset and enable isolation of neural substrates of exposure to different patterns and quantities of ethanol (EtOH). As with humans, a frequently used binge exposure model is thought to produce dependence and affect predominantly corticolimbic brain regions. *in vivo* neuroimaging enables animals models to be examined longitudinally, allowing for each animal to serve as its own control. Accordingly, we conducted 3 magnetic resonance imaging (MRI) sessions (baseline, binge, recovery) to track structure throughout the brains of wild type Wistar rats to test the hypothesis that binge EtOH exposure affects specific brain regions in addition to corticolimbic circuitry. Voxel-based comparisons of 13 EtOH- vs. 12 water- exposed animals identified significant thalamic shrinkage and lateral ventricular enlargement as occurring with EtOH exposure, but recovering with a week of abstinence. By contrast, pretectal nuclei and superior and inferior colliculi shrank in response to binge EtOH treatment but did not recover with abstinence. These results identify brainstem structures that have been relatively underreported but are relevant for localizing neurocircuitry relevant to the dynamic course of alcoholism.

## Introduction

Young adult drinking poses a dire public health issue (Mathurin and Deltenre, 2009) as youth are initiating alcohol use earlier and experiencing more alcohol-related problems than ever before (Gore et al., 2011). As per the Center for Disease Control (CDC) Fact Sheet on Underage Drinking (Centers for Disease Control Prevention, 2018), excessive drinking is responsible for more than 4,300 deaths among underage youth each year. Furthermore, people aged 12–20 years drink 11% of all alcohol consumed in the United States (U.S.) with more than 90% consumed in the form of binge drinking. Based on results of the 2013 National Epidemiologic Surveys on Alcohol and Related Conditions, 15% of young adult males aged 21–25 years drink 10–14 drinks and 13% drink >15 drinks per occasion (Hingson et al., 2017). Similar statistics were reported in 19–20 years old individuals (Monitoring the Future) with 10% of young adults reporting high-intensity drinking of 10+ drinks and 4% reporting >15 drinks per occasion (Patrick and Terry-Mcelrath, 2017). Assuming a young adult U.S. man weighs 180 lbs, 12 drinks can result in blood alcohol levels (BALs) of ~200 mg/dL after 1 h, and 14 drinks in BALs of 230 mg/dL after 3 h of drinking (Miller and Munoz, 2013). Early onset alcohol use is a strong predictor of future alcohol dependence (Hawkins et al., 1997).

Excessive alcohol consumption compromises, among other regions, frontal cortices, thalamus, pons, and cerebellum (e.g., Zahr et al., 2011a; Zahr, 2014; Zahr and Pfefferbaum, 2017; Sullivan et al., 2018). The hippocampus is also susceptible to chronic alcohol exposure (Harding et al., 1997), but generally shows more prominent volume deficits in older alcoholics (e.g., Sullivan et al., 1995; Pfefferbaum et al., 2018).

While animal models are critical for experimental control over factors such as diet and ethanol (EtOH) exposure patterns, pharmacologically relevant BALs can be difficult to achieve (Li et al., 1979, but see Fabio et al., 2014; Jeanblanc et al., 2018). “Dependence,” objectively defined by the presence of withdrawal signs, has be induced with high doses of EtOH, typically via intragastric (French, 2001), intraperitoneal (Pascual et al., 2009, 2014), or vapor (Roberts et al., 2000; Vendruscolo and Roberts, 2014) exposure.

Silver staining of tissue harvested from animals exposed to binge-EtOH (e.g., Majchrowicz model: repetitive daily exposure to ~12 g/kg/day EtOH over 4 days Majchrowicz, 1975; Faingold, 2008) identifies damaged neurons in corticolimbic circuitry including olfactory bulbs, orbital, insular, and piriform cortices, perirhinal and entorhinal cortices, and occasionally in hippocampal CA1, CA2, and CA3 and dentate gyrus (ventral pole) regions (Collins et al., 1996, 1998; Zou et al., 1996; Corso et al., 1998; Crews et al., 2000). A later study, showing pyknotic nuclei with hematoxylin and eosin (H&E) and Fluoro-Jade B stains throughout regions of the corticolimbic circuit shown as injured by silver staining, was interpreted as indicating that binge EtOH exposure, for as little as 2 days, results in necrotic cell death (Obernier et al., 2002). This study spurred an extensive number of reports exploring mechanisms of EtOH-induced degeneration in the hippocampus (e.g., Geisler et al., 1978; Grupp and Perlanski, 1979; Carlen and Corrigall, 1980; Devenport et al., 1981; Roulet et al., 1985; Cadete-Leite et al., 1989; Moghaddam and Bolinao, 1994; Nakano et al., 1996; Nixon and Crews, 2002; Rice et al., 2004; Kelso et al., 2011; Mcclain et al., 2011; Maynard and Leasure, 2013).

By contrast, studies using 2-deoxyglucose, which determine localized changes in glucose metabolism in the central nervous system, show a more widespread signature of EtOH exposure and withdrawal. Evaluation of 2-deoxyglucose utilization administered 10–110 min following exposure to low-to-moderate EtOH (0.25–2g/kg) showed increased glucose utilization in motor and limbic areas, whereas high doses (>1 g/kg) showed decreased local cerebral glucose utilization in several additional structures including thalamus, cerebellum, inferior colliculus, and pons (Eckardt et al., 1988; Grünwald et al., 1993; Williams-Hemby and Porrino, 1994, 1997). Animals withdrawing from EtOH following exposure via the Majchrowicz model (typically 12–18 h after the last dose of EtOH) showed elevations in glucose metabolism not only in the limbic system (i.e., piriform cortex, amygdala, hippocampus), but also in frontal sensorimotor systems, globus pallidus, a number of thalamic and hypothalamic nuclei, cerebellum (flocculus, paraflocculus, vermis, white matter), inferior colliculus, pons, median raphe, and locus coeruleus (Campbell et al., 1982; Eckardt et al., 1986, 1992; Marietta et al., 1986). Indeed, it was noted that while the limbic system generally showed a moderate increase in glucose metabolism relative to controls, mammillary bodies, anterior thalamic nuclei, and cingulate cortices showed more substantial increases during overt withdrawal (Eckardt et al., 1986).

Our previous studies employing a modified Majchrowicz model revealed profound but reversible ventricular enlargement (Zahr et al., 2010b, 2013, 2014b). Using diffusion tensor imaging (DTI), we found decreased mean diffusivity in the thalamus following binge-EtOH treatment that negatively correlated with ventricular expansion (Zahr et al., 2013). Our structural images previously acquired in rats using a human 3T GE magnet did not have the resolution required for careful morphometric assessment of tissue volume changes in response to high EtOH exposure. Equipped with a high field strength 7T animal scanner and voxel-based, rather than region-of-interest (ROI) based morphological evaluation, we now test the hypothesis that extreme binge EtOH exposure to young adult rats modulates multiple brain regions, not limited to corticolimbic circuitry, and predominately affecting thalamic and collicular regions. We further test the hypothesis that these structures would show shrinkage in response to binge EtOH treatment, but recovery with 1 week of abstinence.

## Materials and Methods

### Ethics Statement

All experimental procedures were conducted in accordance with the Guide for the Care and Use of Laboratory Animals of the National Institutes of Health. The Institutional Animal Care and Use Committees at SRI International and Stanford University approved all procedures.

### Animals and Treatment

The study group included 25 male, wildtype Wistar rats (346.2 ± 22.9 g). Male Wistar rats at a weight of 350 g are ~8 weeks old−56 days post-natal (https://www.criver.com/products-services/find-model/wistar-igs-rat?region=3611), corresponding to young adulthood (human equivalent: ages 21–24 years) (Bell et al., 2013). Animals had ad-libitum access to standard laboratory chow and water.

After the baseline scans, 13 rats were assigned to the EtOH group. All animals were fasted overnight and the EtOH group underwent a modified version of the Majchrowicz EtOH-intoxication model (Majchrowicz, 1975) as described in previous studies (Zahr et al., 2010b, 2013, 2014b). Briefly, rats received an initial “loading” dose of 5 g/kg 20% w/v EtOH via oral gavage, then a maximum of 4 g/kg every 8 h (i.e., 6:00, 14:00, and 22:00) for 4 days. On each of the 4 days, animals were weighed and tail vein blood samples were collected ~3.5 h after the 6:00 dose to determine BALs in plasma assayed for alcohol content based on direct reaction with the enzyme alcohol oxidase (Analox Instruments Ltd., UK). After the loading dose, EtOH was administered according to body weight, BALs, and behavioral evidence for intoxication. Control (Con) animals received consistent volumes of drinking water similar to the volumes received by the experimental rats.

Behavior was not assessed in this study. In general, rats exposed to binge EtOH are in good overall health and show remarkable behavioral tolerance to high doses of EtOH. Common signs of intoxication following 4 days of binge EtOH treatment include inactivity and gait disturbances (Zahr et al., 2010b, 2014b). Although evaluated at various time points (e.g., 1–7 days of abstinence) (Zahr et al., 2010b, 2013, 2014b), our binge-EtOH-exposed Wistar rats have not shown overt signs of withdrawal (e.g., tremors, spasticity, “wet-dog” shakes, teeth chattering) (cf., Majchrowicz, 1975).

### MR Scanning Procedures and Data Analysis

#### Schedule

All 25 animals were scanned at baseline (scan 1), after 4 days of treatment (binge (scan 2); ~35 h after the last EtOH dose), and after 7 days of recovery (scan 3). The acute time point was conducted at 35 h to ensure metabolic clearance of EtOH and is roughly equivalent to 1 month of abstinence in humans; 7 days models ~6 months of abstinence in humans (Sengupta, 2013).

#### Anesthesia and Monitoring

Rats were placed on an animal cradle base, including water circulation for body temperature control, over which a rat brain surface coil was secured. At the baseline scan, all animals were anesthetized with 0.1 mg/kg dexmedetomidine (Henry Shein, Melville, NY) by subcutaneous injection. At the binge scan, EtOH-exposed animals could not be fully anesthetized with this dose of dexmedetomidine. Consequently, for binge and recovery scans, animals in both groups were anesthetized with a combination of dexmedetomidine (0.1–0.5 mg/kg, subcutaneous) and ketamine HCl (75 mg/kg, subcutaneous, Henry Shein, Melville, NY). For each rat, temperature and respiration were monitored throughout the ~2 h experiment. All animals received 10cc subcutaneous saline for hydration at the end of the scan.

#### MRI Acquisition

MR data were collected on a Bruker 70/16 US AVANCE III 7.0T system (380 mT/m gradient strength on each (X, Y, and Z) axis, slew rate of 3420 T/m/s) using a Bruker surface coil. A gradient-recalled echo localizer scan was used to position the animals in the scanner and for graphical prescription of the subsequent scans. T2-weighted, high-resolution, TurboRare acquisition sequence: TR = 6000 ms; TE = 33 ms; 0.2 mm isotropic voxels, Matrix = 160 × 160; 2 averages; echo train length = 8; slice thickness = 0.5 mm; interslice gap = 0.5 mm; 32 slices.

#### Image Preprocessing

Preprocessing of each image included removal of noise (Coupe et al., 2008) and inhomogeneity via ANTS 2.1.0 (Tustison et al., 2010). Each image was skull stripped by aligning a template to the scan via symmetric diffeomorphic registration (Avants et al., 2008) and the resulting deformation map was applied to the brain mask of the template. Image inhomogeneity correction was repeated on skull-stripped images. Bias-corrected, skull-stripped images were rigidly aligned to a template via ANTS 2.1.0 and used as input for further analysis.

#### Quantification of Structural Changes

For each animal, the intensity profile of scan 1 (baseline) was matched to the intensity profile of scan 2 (binge) and the resulting images were non-rigidly registered using ANTS 2.1.0 (Avants et al., 2011). The resulting deformation map was transformed into a log Jacobian determinant map (i.e., Jacobian map) to measure structural changes between the first two scans (Ashburner and Friston, 2003; Riddle et al., 2004; Hua et al., 2008).

To align the Jacobian maps of all animals to a common coordinate space, baseline scans from all animals were registered to one another using group-wise, non-rigid registration (Joshi et al., 2004). This process resulted in a common template specific to the baseline scan. The Jacobian maps were aligned to the template according to the deformation maps associated with the group-wise registration. Focusing on bilateral structural changes, the resulting Jacobian maps were bilaterally averaged with respect to the central sagittal slice by averaging the two values from the right and left hemispheres at each voxel.

To measure structural changes between scan 2 (binge) and scan 3 (recovery), the process of computing Jacobian maps and aligning them to template space was repeated. Note the resulting template, created by applying group-wise registration to all scan 2 (binge) images, was thus different from the scan 1 (baseline) template. This approach was chosen over aligning images of all times points to a single template in order to reduce the complexity associated with group-wise registration and the risk of introducing errors into the analysis.

To analyze detected clusters in a common space, cluster locations were transformed to the digital Paxinos-Watson (PW) rat brain atlas (Nie et al., 2013) (labels 43 regions: 28 bilateral, and 15 midline) by non-rigid registration of the templates associated with scans 1 and 2 to the atlas. For full disclosure, we include similar results using the Waxholm Space atlas of the Sprague Dawley (WHS SD) rat brain (Papp et al., 2014) (labels 74 regions).

### Statistical Analysis

Simple *t*-tests compared the two groups on weights and BALs. For image analysis, significant differences in tissue expansion or shrinkage between the EtOH and control group were examined on the baseline Jacobian maps by applying one-tailed *t*-tests to each voxel in the right hemisphere of the bilaterally-averaged Jacobian maps. We expected tissue shrinkage between baseline and binge, and tissue expansion between binge and recovery; we expected CSF expansion between baseline and binge, and CSF reduction between binge and recovery. These tests carried out by the Permutation Analysis of Linear Model package (PALM, version alpha109) (Winkler et al., 2014) identified significant voxels with p < 0.01. Results were corrected for multiple comparisons using AFNI 3dClustSim V17.3.09 (Forman et al., 1995) with a false-positive control rate of α = 0.05 as the threshold for the minimum size of a cluster of significance. For the 2-group (EtOH vs. control) comparison, scan 1 vs. 2 the number of contiguous voxels denoting a cluster size of significance was 607; for scans 2 vs. 3 the number of contiguous voxels was 614 (for within group comparisons: EtOH scans 1–2, 595; EtOH scans 2–3, 607; control scans 1–2, 584; control scans 2–3, 605). For visualization purposes, the cluster of voxels that reached significance was directly mapped to the left-hemisphere.

Within each group, significant tissue shrinkage or expansion was detected similarly using one-tailed *t*-tests (*p* < 0.01) in combination with multiple-comparison correction applied to the Jacobian maps of all animals using the previously-defined thresholds. Between-group and within-group comparisons were repeated for the Jacobian maps computed between the binge 2 and recovery 3 scans.

Finally, to analyze all detected clusters in a common space, the cluster locations were transformed into the PW atlas space by non-rigidly registering the templates associated with scans 1 and 2 to the atlas. The resulting deformation maps were applied to the binary masks of the clusters to align to the PW atlas space. The percentage of each ROI (as provided by the PW atlas) that overlapped with the aligned binary masks was then computed. We chose a threshold of 10% expansion or shrinkage as relevant to discuss based on previous studies: up to 10% tissue expansion was observed in the occipital cortex in response to learning-associated plasticity (Patton et al., 2018); in a study of radiation-induced ventilation changes calculated with Jacobian maps, a 10% volume expansion threshold was similarly used (Lövdén et al., 2013).

## Results

EtOH animals were exposed to a cumulative 4 days sum of 31.0 ± 2.0 g/kg EtOH (range 27–34 g/kg), while control animals received a cumulative 4 days sum of 35 g/kg water. Across the 4 days of exposure, average BALs in the EtOH group were 289.0 ± 13.7 mg/dL and peak BALs reached 317.8 ± 24.3 mg/dL. Weight did not distinguish the 2 groups of animals at the baseline scan (344.3 ± 27.5 g control group; 348.0 ± 18.7 g EtOH group, *p* = 0.70) or the 2 follow-up scans (binge scan: 300.7 ± 28.5 control, 218.8 ± 22.7 EtOH, *p* = 0.08; recovery scan: 357.0 ± 36.3 control, 331.7 ± 27.8 EtOH, *p* = 0.06).

## Results From PW Atlas

### Between-Group Differences

Table 1 presents between group (EtOH vs. control) differences in voxels identified in between scan comparisons: baseline scan 1 vs. binge scan 2 and binge scan 2 vs. recovery scan 3. While all identified changes are included, only those above 10% are highlighted and discussed.

**Table 1:** Between group analysis using PW atlas.

	**ROI vol**	**Time 1–2**				**Time 2–3**				**Overall**
**ROI Name**		**Expand vol**	**Expand %**	**Shrink vol**	**Shrink %**	**Expand vol**	**Expand %**	**Shrink vol**	**Shrink %**	
**GRAY MATTER**										
**Cortical regions**										
Somatosensory	7,858	n.d.	n.d.	n.d.	n.d.	n.d.	n.d.	3	0.24%	−0.24%
Motor	4,662	73	1.70%	n.d.	n.d.	85	1.91%	n.d.	n.d.	3.61%
Auditory	1,457	n.d.	n.d.	n.d.	n.d.	n.d.	n.d.	21	3.06%	−3.06%
Temporal association	597	n.d.	n.d.	n.d.	n.d.	n.d.	n.d.	2	0.34%	−0.34%
Piriform	3,016	n.d.	n.d.	n.d.	n.d.	37	1.41%	n.d.	n.d.	1.41%
Cingulate	2,328	56	2.41%	n.d.	n.d.	21	0.90%	n.d.	n.d.	3.31%
Prelimbic	1,205	91	7.55%	n.d.	n.d.	98	8.13%	n.d.	n.d.	**15.68%**
Infralimbic	342	n.d.	n.d.	n.d.	n.d.	60	**17.54%**	n.d.	n.d.	**17.54%**
Dorsal peduncular	181	n.d.	n.d.	n.d.	n.d.	12	6.63%	n.d.	n.d.	6.63%
Hippocampus (dorsal)	4,507	132	3.24%	n.d.	n.d.	345	8.39%	287	6.37%	5.25%
Hippocampus (ventral)	3,233	1	0.03%	n.d.	n.d.	67	1.58%	120	3.71%	−2.10%
Caudate	4,809	61	1.14%	6	0.12%	115	2.52%	89	1.39%	2.15%
Nucleus accumbens shell	447	n.d.	s	n.d.	n.d.	38	7.64%	n.d.	n.d.	7.64%
Nucleus accumbens core	558	n.d.	n.d.	n.d.	n.d.	129	**23.02%**	n.d.	n.d.	**23.02%**
Globus pallidus	552	n.d.	n.d.	n.d.	n.d.	4	0.90%	n.d.	n.d.	0.90%
Septum	1,275	n.d.	n.d.	n.d.	n.d.	1	0.08%	n.d.	n.d.	0.08%
Thalamus (vpl/vpm)	487	n.d.	n.d.	3	0.62%	n.d.	n.d.	n.d.	n.d.	−0.62%
Geniculate nuclei	527	n.d.	n.d.	2	0.62%	53	8.40%	n.d.	n.d.	7.78%
Thalamus (central)	656	n.d.	n.d.	139	**20.66%**	164	**25.30%**	n.d.	n.d.	4.64%
Anterior pretectal nuclei	67	n.d.	n.d.	2	**15.90%**	n.d.	n.d.	n.d.	n.d.	**−15.90%**
Superior colliculus	1,635	n.d.	n.d.	272	**16.45%**	n.d.	n.d.	n.d.	n.d.	**−16.45%**
Inferior colliculus	1,500	n.d.	n.d.	181	**12.32%**	n.d.	n.d.	n.d.	n.d.	**−12.32%**
Substantia nigra	265	n.d.	n.d.	5	1.10%	n.d.	n.d.	n.d.	n.d.	−1.10%
Periaquaductal gray	1,820	n.d.	n.d.	18	0.99%	n.d.	n.d.	n.d.	n.d.	−0.99%
Pontine reticular nuclei	1,013	n.d.	n.d.	37	3.65%	n.d.	n.d.	n.d.	n.d.	−3.65%
Reticular formation	6,171	n.d.	n.d.	68	1.10%	n.d.	n.d.	n.d.	n.d.	−1.10%
Cerebellum	1,1619	n.d.	n.d.	335	2.88%	n.d.	n.d.	n.d.	n.d.	−2.88%
Unlabeled	9,4035	878	0.93%	4,233	4.50%	1,640	1.74%	585	0.62%	−2.45%
**WHITE MATTER**										
Corpus collosum	6,408	143	2.23%	n.d.	n.d.	n.d.	n.d.	453	7.07%	−4.84%
**Cerebrospinal Fluid**										
Above frontal cortical regions	483	76	**15.73%**	n.d.	n.d.	n.d.	n.d.	n.d.	n.d.	**15.73%**
Ventricles	253	221	**87.35%**	n.d.	n.d.	n.d.	n.d.	220	**86.96%**	**0.40%**

*n.d, not detected. For this and all subsequent tables, only regions showing 10% change are in bold and discussed*.

**Figure 1:**
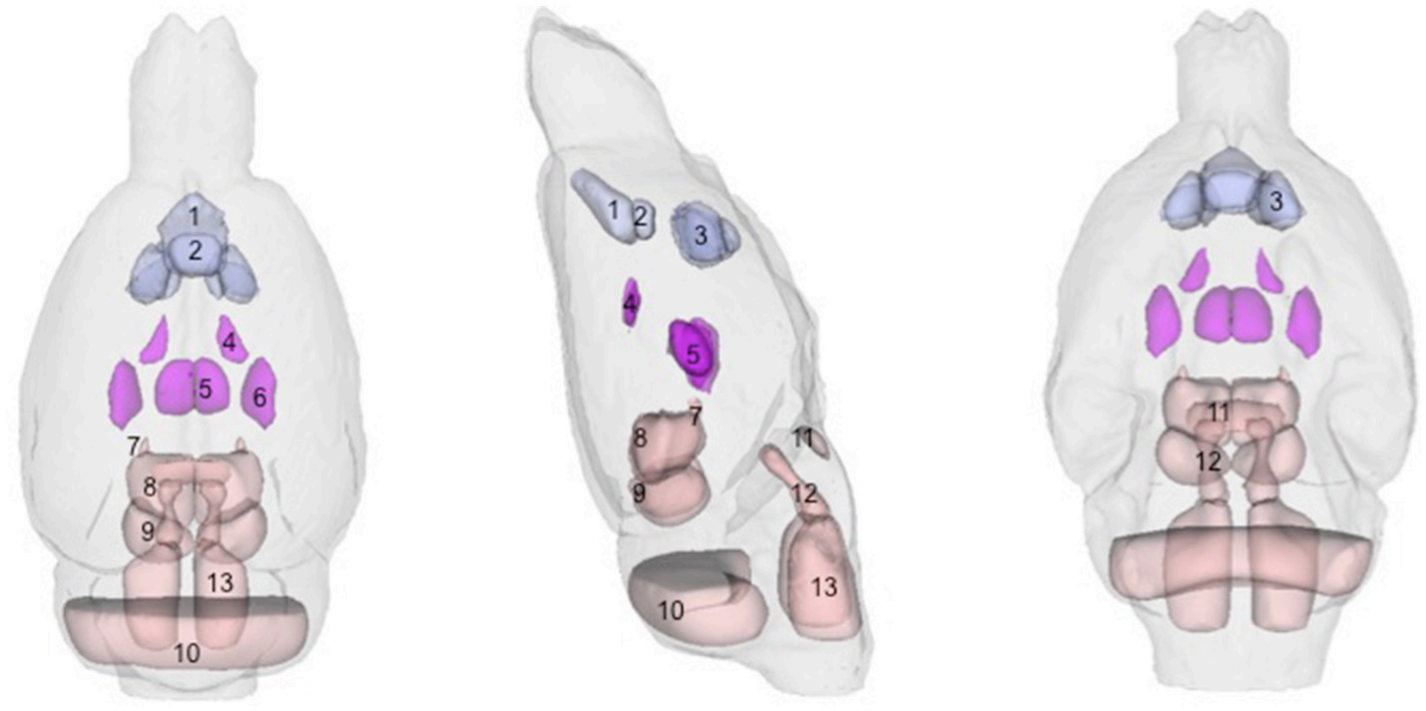
Regions-of-interest (ROIs) identified via the Paxinos-Watson (PW) digital atlas to show at least 10% volume change between groups over the course of the experiment (refer to Table 1): (1) infralimbic cortex, (2) prelimbic cortex, (3) nucleus accumbens (core and shell), (4) lateral ventricles, (5) central thalamus, (6) VPL/VPM (ventral posterior lateral and medial) thalamus, (7) anterior pretectal nuclei, (8) superior colliculus, (9) inferior colliculus. Also labeled, (10) cerebellum, (11) pontine nuclei, (12) pontine reticular nuclei, (13) reticular nuclei of the pons. ROIs in purple indicate reversible effects of EtOH exposure and recovery; ROIs in pink indicate enduring shrinkage; ROIs in light blue indicate enduring expansion.

**Figure 2:**
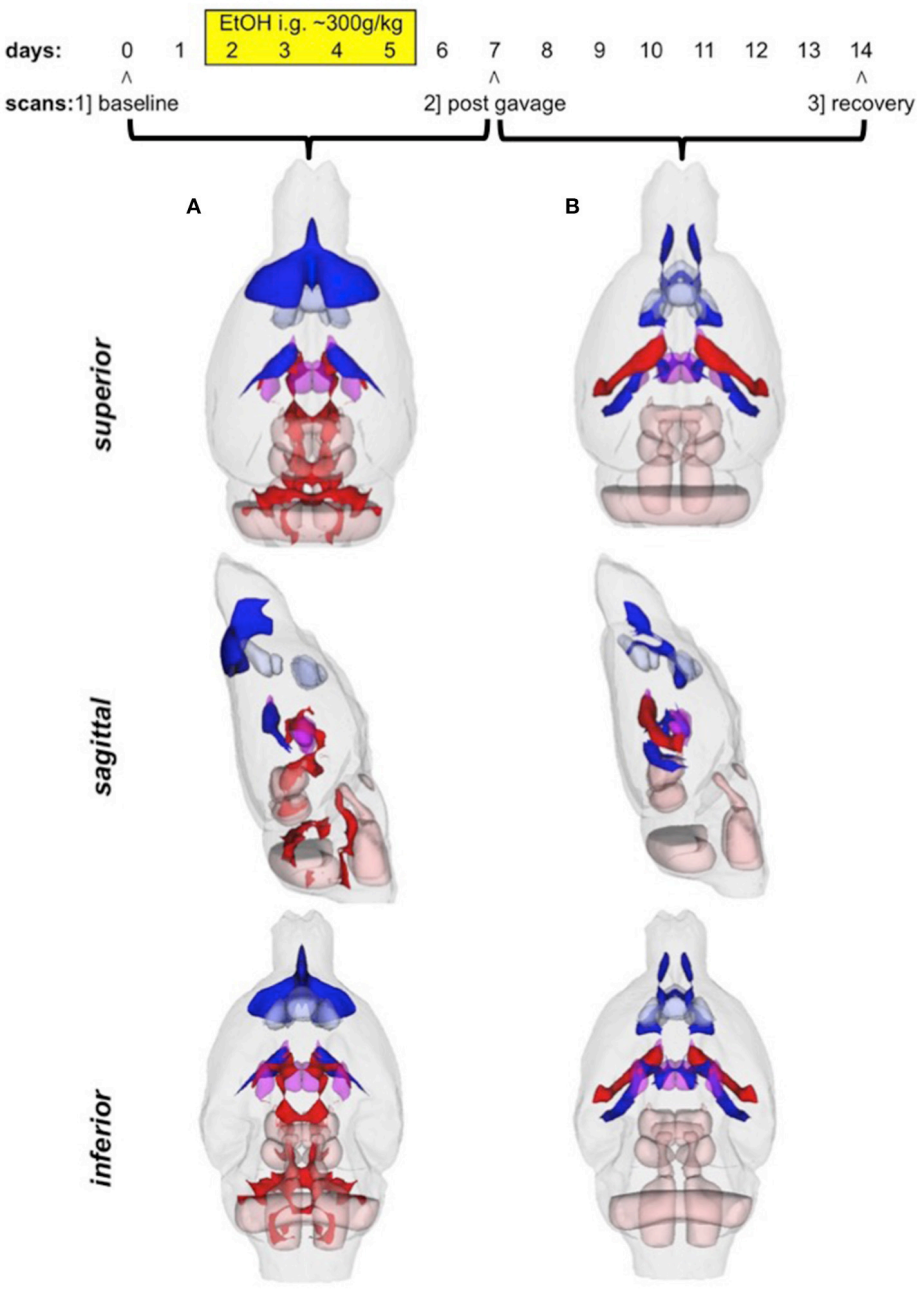
**(A)** Voxels showing expansion (blue) and shrinkage (red) between baseline scan 1 and binge scan 2 superimposed on Figure 1 ROIs; **(B)** voxels showing expansion (blue) and shrinkage (red) between binge scan 2 and recovery scan 3 superimposed on Figure 1 ROIs.

There were several patterns of changes identified between the EtOH and control groups. Figure 1 labels significant ROIs identified by the PW atlas with changes color-coded as noted below; Figure 2A shows expansion (blue) and shrinkage (red) between baseline scan 1 and binge scan 2 superimposed on Figure 1 ROIs; and Figure 2B shows expansion (blue) and shrinkage (red) between binge scan 2 and recovery scan 3 superimposed on Figure 1 ROIs.

### Reversible Changes (Purple ROIs)

The ventricles showed an 87% expansion with binge EtOH exposure that was reversed by 1 week of abstinence (87% shrinkage). The thalami showed an opposite pattern: they shrank by 21% during binge EtOH exposure but showed volume recovery (25% expansion) after a week of abstinence.

### Enduring Tissue Shrinkage (Red ROIs)

The anterior pretectal nuclei (−16%), superior colliculi (−16%), and inferior colliculi (−12%) shrank with binge EtOH exposure but did not show volume recovery during the week of abstinence.

### Enduring Tissue Expansion (Blue ROIs)

Two cortical regions showed enduring expansion: prelimbic areas expanded at both time points for a total of 16% expansion; infralimbic regions expanded (18%) during the week of abstinence. Similarly, the nucleus accumbens core showed 23% expansion during the week of abstinence.

### Enduring Fluid Expansion (Blue ROIs)

The cerebrospinal fluid (CSF) above frontal regions non-reversibly expanded following binge EtOH treatment.

### Within-Group Differences

Table 2 presents within-EtOH group differences in voxels identified in between baseline scan 1 vs. binge scan 2 and binge scan 2 vs. recovery scan 3. These comparisons within the EtOH group identified a similar pattern: ventricles were reversibly enlarged; pretectal nuclei, superior and inferior colliculi shrank; and prelimbic and infralimbic cortices and the CSF above frontal regions expanded. This within-group comparison showed thalamic expansion only at the recovery scan 3 (i.e., thalamic shrinkage was not noted at the binge scan 2).

**Table 2:** Within EtOH group analysis using PW atlas.

	**ROI vol**	**Time 1–2**				**Time 2–3**				**Overall**
**ROI Name**		**Expand vol**	**Expand %**	**Shrink vol**	**Shrink %**	**Expand vol**	**Expand %**	**Shrink vol**	**Shrink %**	
**GRAY MATTER**										
**Cortical regions**										
Somatosensory	7,858	14	0.11%	319	4.18%	52	0.62%	n.d.	n.d.	−3.45%
Motor	4,662	n.d.	n.d.	n.d	n.d	58	**11.96%**	n.d.	n.d.	**11.96%**
Visual	3,344	n.d.	n.d.	342	**10.38%**	n.d.	n.d.	n.d.	n.d.	**−10.38%**
Auditory	1,457	n.d.	n.d.	n.d.	n.d.	n.d.	n.d.	42	3.86%	−3.86%
Parietal association	294	n.d.	n.d.	10	3.11%	n.d.	n.d.	n.d.	n.d.	−3.11%
Temporal association	597	n.d.	n.d.	20	5.36%	n.d.	n.d.	5	0.84%	−6.20%
Rhinal	3,397	1	0.20%	387	**12.17%**	n.d.	n.d.	26	0.77%	**−12.74%**
Insular	1,968	n.d.	n.d.	140	6.66%	n.d.	n.d.	n.d.	n.d.	−6.66%
Piriform	3,016	n.d.	n.d.	2	0.18%	32	1.25%	45	1.07%	0.00%
Cingulate	2,328	92	3.95%	4	0.17%	48	2.06%	n.d.	n.d.	5.84%
Retrospenial	3,539	n.d.	n.d.	451	**12.74%**	n.d.	n.d.	n.d.	n.d.	**−12.74%**
Prelimbic	1,205	162	**13.44%**	8	0.66%	149	**12.37%**	n.d.	n.d.	**25.15%**
Infralimbic	342	n.d.	n.d.	n.d.	n.d.	61	**17.84%**	n.d.	n.d.	**17.84%**
Dorsal peduncular	181	n.d.	n.d.	n.d.	n.d.	28	**15.47%**	n.d.	n.d.	**15.47%**
Hippocampus (dorsal)	4,507	184	4.50%	47	1.07%	n.d.	n.d.	n.d.	n.d.	3.43%
Hippocampus (ventral)	3,233	225	7.35%	71	3.29%	405	**10.21%**	284	6.93%	7.33%
Amygdala	1,359	18	0.70%	21	0.88%	393	**11.89%**	180	4.95%	6.76%
Caudate	4,809	214	4.72%	98	2.12%	n.d.	n.d.	n.d.	n.d.	2.60%
Nucleus accumbens shell	447	n.d.	n.d.	74	**13.97%**	39	1.05%	124	1.44%	**−14.36%**
Nucleus accumbens core	558	n.d.	n.d.	61	**13.62%**	45	**10.12%**	n.d.	n.d.	−3.50%
Septum	1,275	147	**11.53%**	80	6.27%	121	**20.75%**	n.d.	n.d.	**26.00%**
Thalamus (vpl/vpm)	487	n.d.	n.d.	4	0.82%	12	0.94%	n.d.	n.d.	0.12%
Geniculate nuclei	527	n.d.	n.d.	n.d.	n.d.	n.d.	n.d.	n.d.	n.d.	0.00%
Thalamus (central)	656	n.d.	n.d.	1	0.15%	127	**22.26%**	n.d.	n.d.	**22.10%**
Hypothalamus	1,690	5	0.49%	184	**11.00%**	246	**37.44%**	n.d.	n.d.	**26.93%**
Anterior pretectal nuclei	67	n.d.	n.d.	21	**30.08%**	3	0.46%	n.d.	n.d.	**−29.62%**
Superior colliculus	1,635	n.d.	n.d.	259	**15.47%**	n.d.	n.d.	n.d.	n.d.	**−15.47%**
Inferior colliculus	1,500	n.d.	n.d.	228	**15.38%**	n.d.	n.d.	n.d.	n.d.	**−15.38%**
Substantia nigra	265	7	5.81%	12	4.19%	96	5.72%	n.d.	n.d.	7.34%
Raphe nuclei	479	n.d.	n.d.	2	0.42%	13	6.62%	n.d.	n.d.	6.20%
Pontine reticular nuclei	1,013	n.d.	n.d.	67	6.61%	n.d.	n.d.	n.d.	n.d.	−6.61%
Reticular formation	6,171	n.d.	n.d.	101	1.64%	n.d.	n.d.	n.d.	n.d.	−1.64%
Cerebellum	1,1619	58	0.50%	n.d.	n.d.	n.d.	n.d.	n.d.	n.d.	0.50%
Unlabeled	9,4035	4,845	5.15%	4,829	5.14%	3,244	3.45%	605	0.64%	2.82%
**WHITE MATTER**										
Corpus collosum	6,408	223	3.48%	503	7.85%	3	0.05%	397	6.20%	**−10.52%**
Internal capsule	2,202	n.d.	n.d.	36	1.63%	16	0.73%	n.d.	n.d.	−0.91%
Mammilothalamic tract	655	56	8.55%	4	0.61%	256	**16.03%**	n.d.	n.d.	**23.96%**
**CEREBROSPINAL FLUID**										
Above frontal regions	483	91	**18.95%**	n.d.	n.d.	n.d.	n.d.	n.d.	n.d.	**18.95%**
Ventricles	253	248	**98.02%**	n.d.	n.d.	n.d.	n.d.	225	88.93%	9.09%
Above pons	459	161	**35.08%**	n.d.	n.d.	n.d.	n.d.	n.d.	n.d.	**35.08%**

*n.d, not detected. only regions showing 10% change are in bold and discussed*.

In addition, the within-EtOH-group comparison showed reversible shrinkage of the hypothalamus and enduring tissue shrinkage of the following structures: corpus callosum (−11%), visual (−11%), rhinal (−13%), and retrosplenial (−13%) cortices, and nucleus accumbens shell (−14%). Several regions showed enduring expansion: septum (26%), mammilothalamic tracts (24%), and motor (12%) and dorsal peduncular (15%) cortices.

Table 3 presents within control group comparisons. The geniculate nuclei (17%) and thalami (24%) expanded between binge scan 2 and recovery scan 3.

**Table 3:** Within control group analysis using PW atlas.

	**ROI vol**	**Time 1–2**				**Time 2–3**				**Overall**
**ROI Name**		**Expand vol**	**Expand %**	**Shrink vol**	**Shrink %**	**Expand vol**	**Expand %**	**Shrink vol**	**shrink %**	
**GRAY MATTER**										
**Cortical regions**										
Somatosensory	7,858	n.d.	n.d.	n.d.	n.d.	n.d.	n.d.	35	0.33%	−0.33%
Motor	4,662	n.d.	n.d.	n.d.	n.d.	n.d.	n.d.	37	0.51%	−0.51%
Visual	3,344	n.d.	n.d.	n.d.	n.d.	n.d.	n.d.	17	0.51%	−0.51%
Rhinal	3,397	n.d.	n.d.	n.d.	n.d.	n.d.	n.d.	16	1.57%	−1.57%
Insular	1,968	n.d.	n.d.	n.d.	n.d.	n.d.	n.d.	2	0.10%	−0.10%
Piriform	3,016	n.d.	n.d.	n.d.	n.d.	n.d.	n.d.	34	0.64%	−0.64%
Retrospenial	3,539	n.d.	n.d.	n.d.	n.d.	n.d.	n.d.	1	0.03%	−0.03%
Hippocampus (dorsal)	4,507	n.d.	n.d.	n.d.	n.d.	72	2.17%	148	3.53%	−1.36%
Hippocampus (ventral)	3,233	n.d.	n.d.	n.d.	n.d.	6	0.26%	180	4.56%	−4.30%
Amygdala	1,359	n.d.	n.d.	n.d.	n.d.	n.d.	n.d.	1	0.40%	−0.40%
Geniculate nuclei	527	n.d.	n.d.	n.d.	n.d.	103	**17.56%**	3	1.06%	**16.49%**
Thalamus (central)	656	n.d.	n.d.	n.d.	n.d.	167	**23.65%**	n.d.	n.d.	**23.65%**
Superior colliculus	1,500	n.d.	n.d.	n.d.	n.d.	n.d.	n.d.	11	0.61%	−0.61%
Inferior colliculus	1,635	n.d.	n.d.	n.d.	n.d.	n.d.	n.d.	22	1.40%	−1.40%
Cerebellum	11,619	164	1.41%	n.d.	n.d.	n.d.	n.d.	13	0.11%	1.30%
Unlabeled	94,035	711	0.76%	n.d.	n.d.	819	0.87%	756	0.80%	0.82%
**WHITE MATTER**										
internal capsule	2,202	n.d.	n.d.	n.d.	n.d.	2	0.09%	n.d.	n.d.	0.09%

*n.d, not detected. only regions showing 10% change are in bold and discussed*.

## Results From WHS SD Atlas

In addition to the PW atlas, we used the WHS SD rat brain atlas (Papp et al., 2014) to identify regional difference between and within groups (Supplementary Tables 1–3). These additional results highlight overlaps in the regions identified by the two atlases in response to binge EtOH treatment and recovery and also indicate differences. In the between-group comparisons, pretectal regions (−11%), superior colliculus deep layers (−19%), and inferior colliculus commissure (−26%) shrank and the thalamus showed reversible shrinkage with binge EtOH exposure, consistent with findings based on the PW atlas. This higher resolution atlas additionally showed shrinkage of the facial nerve (−63%); reversible expansion with overall shrinkage in hippocampal cornu ammonis 2 (−21%) and cornu ammonis 3 (−12%); and expansion in accessory olfactory bulb (17%) and bed nucleus of the stria terminalis (16%).

## Discussion

The present study, the first to use a voxel- rather than a ROI- based approach to evaluate global, morphological brain changes in response to binge-EtOH treatment and recovery in rats shows a more extensive brain response to binge-EtOH exposure than have cross-sectional, histological studies. As previously reported, the ventricles showed reversible enlargement and the thalamus showed reversible shrinkage (Zahr et al., 2013). We now describe enduring shrinkage of pretectal nuclei, and superior and inferior colliculi as identified by both the PW and WHS SD atlases.

### Limitations

For a clear discussion of the results, it is critical to recognize the limitations of this study. Although a high field-strength (i.e., 7T) resulted in the acquisition of high-resolution images (0.2 mm isotropic vs. 25 × 0.25 × 0.7 mm3), automated quantification is limited by atlas resolution. We focused on PW-atlas-derived results because resolution (i.e., 068 × 0.091 × 0.58 mm3) was closer than the WHS SD atlas (0.078 mm isotropic) to that achieved by our acquisition.

With respect to accuracy, flaws in PW-atlas segmentation are perhaps most obvious in the labeling of cerebellum (Figure 1, #10) and pontine structures (Figure 1, #11–13). The digital PW atlas ignores the anterior half of the cerebellum and superior portions of pontine structures. This labeling inaccuracy results in a fewer percentage of affected voxels in the cerebellum and pons than would be expected based on visual inspection (Figure 2).

Along a similar vein, qualitative (i.e., manual) identification of regions based on a printed version of PW atlas (Paxinos and Watson, 1998) demonstrated that large parts of the thalamus, unlabeled by either digital atlas, were significantly affected including dorsolateral geniculate nuclei and reticular, paraventricular, anterodorsal, ventral anterior, ventral lateral, ventral medial, and parafasicular thalamic nuclei.

Further, the emphasis of the two atlases was different: in the PW atlas, cortical regions were sub-segmented whereas in the WHS SD atlas, hippocampal regions were sub-segmented. The prominence of the hippocampus in the WHS SD atlas, for example, overshadowed the ventricles, which were hardly visible; thus, quantification showed no effects of binge-ETOH treatment on the ventricles, but showed considerable enlargement of the adjacent tissue of cornu ammonis 1. In fact, both visual inspection and the PW atlas indicate this quantification was inaccurate (ventricles expanded, portions of the hippocampus shrank).

Finally, another indication of the limitations of the atlases is that while there was some overlap in identified regions, they also highlight different areas affected by binge EtOH exposure and abstinence. The PW atlas identified expansion of CSF above frontal regions and of tissue in prelimbic and infralimbic cortices and nucleus accumbens. The WSH SD atlas showed tissue expansion in accessory olfactory bulb and bed nucleus of the stria terminalis, and shrinkage of the facial nerve and hippocampal cornu ammonis 2 and 3.

Neither atlas showed significant effects on globus pallidus or locus coeruleus, although these structures were often reported as affected in 2-deoxyglucose studies (Campbell et al., 1982; Eckardt et al., 1986, 1992; Marietta et al., 1986). For the globus pallidus, this is unlikely due to inaccurate segmentation, because the Jacobians showed few effects in the vicinity of this ROI. By contrast, neither atlas labeled the locus coeruleus, but visual inspection of the within-EtOH-group comparison showed a prominent volume deficit in this area.

### Results in Context

A voxel-based approach identified significant changes in response to binge EtOH treatment throughout the rat brain that parallel regions identified in human alcoholism (e.g., Zahr et al., 2011a; Zahr, 2014; Zahr and Pfefferbaum, 2017; Sullivan et al., 2018). In particular, the thalamus and colliculi feature significantly in the literature on Korsakoff syndrome and Wernicke's encephalopathy (Sullivan and Pfefferbaum, 2009; Jung et al., 2012), sequelae of chronic alcoholism attributable to dietary depletion of thiamine (Zahr et al., 2011a,b).

In rats, lesions resulting from thiamine deficiency (i.e., pyrithiamine treatment) localized to the medial thalamus are associated with impaired spatial learning (Mair et al., 1991; see also Vedder et al., 2015). Rats with direct lesions to the thalamic internal medullary lamina (Mair and Lacourse, 1992) or anterior thalamus (Célérier et al., 2000) similarly show deficits in a spatial memory. This evidence for an association between specific thalamic nuclei and working memory, together with evidence that rats exposed to 4 months of EtOH show deficits in spatial working memory (Santín et al., 2000), implies that EtOH-induced changes to thalamic nuclei (as identified herein) contribute to a spatial memory deficits.

Lesions to the superior colliculus in rats are associated with hyperactivity (Foreman et al., 1978; Pope and Dean, 1979). Exposure to alcohol in healthy male volunteers is associated with hyperactivity (Marinkovic et al., 2000) as is withdrawal from alcohol (Erstad and Cotugno, 1995). Psychomotor hyperactivity in Wernicke's encephalopathy may be linked to superior colliculus compromise (Kleinert-Altamirano and Juarez-Jimenez, 2014), but a direct relationship between EtOH-induced hyperactivity and changes to the superior colliculus has yet to be demonstrated.

The current results comport with previous MRI studies evaluating the effects of EtOH on rodent brains, though none have used the Majchrowicz model and none were performed in adult, wild type rats. For example, a study employing voluntary drinking in alcohol-preferring (AA) rats and manganese-enhanced MRI found intensity changes in the somatosensory cortex, accumbens shell, ventral thalamus, lateral hypothalamus, ventral hippocampus, and superior colliculus (Dudek et al., 2015). Another MRI study reported alterations in the volumes of neocortex, striatum, thalamus, hypothalamus, and hippocampus of adolescent rats exposed to intermittent EtOH vapor (Gass et al., 2014). Orbitofrontal cortices, cerebellum, thalamus, internal capsule, and corpus callosum were modified in adolescent mice given intermittent EtOH via intragastric gavage (Coleman et al., 2014). In summary, the pattern of regions modulated by forced EtOH exposure in rodents and identified by both 2-deoxyglucose and MRI studies include not only the corticolimbic circuitry, so often studied in this context, but also the thalamus, colliculi, cerebellum, and pons.

### Speculation About Mechanisms

At least four patterns were observed: (1) reversible shrinkage (thalamus) and expansion (ventricles); enduring tissue (2) shrinkage or (3) expansion, and (4) enduring fluid expansion. These patterns suggest that different mechanisms may be triggered by binge-EtOH treatment depending on the brain region or cell type considered.

Reversible ventriculomegaly and concurrent reversible shrinkage of thalamic nuclei in response to binge-EtOH has led us to speculate (Zahr et al., 2013) that EtOH causes intracellular water extravasation leading to shrinkage of brain cells and their process with parallel expansion of ventricular volume (cf., Streitbürger et al., 2012). EtOH affects water movement (Champion et al., 1975; Klemm, 1998), which can include loss of intracellular water (Pollock and Arieff, 1980) and brain cell shrinkage (Cserr et al., 1987). Two *in vitro* studies (Sripathirathan et al., 2009; Collins et al., 2013) and one *in vivo* experiment (Tajuddin et al., 2013) have now demonstrated that expression of the water channel protein aquaporin-4 (AQP4) can be modulated by exposure to EtOH, a mechanism possibly responsible for fluid redistribution caused by exposure to high EtOH levels.

Enduring tissue shrinkage may be caused by compromise of the brain's normal energy utilization by binge EtOH. Altered energy utilization (as implicated by 2-deoxyglucose studies) (e.g., Eckardt et al., 1992) can lead to cellular degeneration (Duan et al., 2014; Di Domenico et al., 2017) and consequently volume shrinkage. Further, in a model of thiamine deficiency, which affects energy expenditure (Liu et al., 2014b), we found similar brain regions affected, notably the thalamus and colliculi (Zahr et al., 2014a). While our previous work demonstrates that binge EtOH exposure does not quantifiably modify peripheral thiamine levels (Zahr et al., 2010a), it is nonetheless possible that subclinical nutritional deficiencies in response to binge EtOH treatment can affect the energetic balance of the brain.

More difficult to explain are the regions—for example, prelimbic and infralimbic cortices and nucleus accumbens—that showed expansion during the week of recovery. Possibly, this is EtOH-related edema (Liu et al., 2014a); alternatively, these regions may be susceptible to hypoxia and subsequent gliosis (Thanos et al., 2016), which might putatively contribute to volume expansion.

## Conclusions

While mechanistic explanations are limited to conjecture, the current study highlights brain regions that are understudied in alcoholism. Indeed, a Pubmed search for “alcohol^*^ and ethanol and hippocampus” limited to “other animals” identifies 1421 manuscripts, while a similar search for “alcohol^*^ and ethanol and thalamus” produces only 133 results and that for “alcohol^*^ and ethanol and colliculus” returns only 40 manuscripts. This is surprising given that human studies have traditionally found fewer effects of AUD on the hippocampus than on the thalamus (Sullivan et al., 1995; Harding et al., 1997) and because of evidence that mnemonic deficits in alcoholism are more likely related to thalamic integrity (Fama et al., 2014; Pitel et al., 2015).

In conclusion, this voxel-based morphometric analysis identified brain volumes significantly modulated by binge-EtOH exposure and subsequent abstinence. Findings from this longitudinal study, indicating that some changes are reversible and some are not expressed until after the week of abstinence (e.g., infralimbic cortex) are not possible using cross-sectional study. Significantly, this analysis highlights the thalamus and colliculi as regions that are clearly modified by exposure to EtOH. Still unresolved are the mechanisms that underlie EtOH-related volumetric changes within and outside the limbic systemand how volumetric changes influence neural connectivity and function.

## Author Contributions

QZ did the data analysis using MIPAV and other tools. MF helped with confirming anatomical boundaries. AP designed the experiment. ES helped design the experiment and write the manuscript. KP is QZ's advisor. NZ worked with QZ to figure out best way to analyze and present data and wrote the manuscript.

### Conflict of Interest Statement

The authors declare that the research was conducted in the absence of any commercial or financial relationships that could be construed as a potential conflict of interest.
