# Supplementary material for: Jacobian Maps Reveal Under-reported Brain Regions Sensitive to Extreme Binge Ethanol Intoxication in the Rat
Source: Front Neuroanat. 2018 Dec 11;12:108. doi: 10.3389/fnana.2018.00108 (PMC6297262; doi:10.3389/fnana.2018.00108)
Supplement: Supplementary file 1 [file Data_Sheet_1.PDF]

Supplementary Table 1. Between group analysis using WHS SD atlas

ROI Name	ROI vol	time 1 - 2				time 2 - 3				Overall
		expand vol	expand %	shrink vol	shrink %	expand vol	expand %	shrink vol	shrink %	
**Gray Matter**										
accessory olfactory bulb (glomerular layer)	151	22	**14.57%**	n.d.	n.d.	4	2.65%	n.d.	n.d.	**17.22%**
olfactory bulb (glomerular layer)	1359	6	0.44%	n.d.	n.d.	65	4.78%	n.d.	n.d.	5.22%
olfactory bulb	15900	679	4.27%	n.d.	n.d.	653	4.11%	n.d.	n.d.	8.38%
neocortex	66108	2025	3.06%	n.d.	n.d.	451	0.68%	109	0.16%	3.58%
hippocampal formation										
cornu ammonis 1	4382	226	5.16%	n.d.	n.d.	644	**14.70%**	316	7.21%	**12.64%**
cornu ammonis 2	382	76	**19.90%**	n.d.	n.d.	1	0.26%	157	**41.10%**	**-20.94%**
cornu ammonis 3	3705	605	**16.33%**	7	0.19%	60	1.62%	1092	**29.47%**	**-11.71%**
dentate gyrus	4570	51	1.12%	45	0.98%	252	5.51%	105	2.30%	3.35%
fasciola cinereum	479	3	0.63%	n.d.	n.d.	18	3.76%	4	0.84%	3.55%
subiculum	2273	n.d.	n.d.	n.d.	n.d.	1	0.04%	n.d.	n.d.	0.04%
striatum	11354	n.d.	n.d.	9	0.08%	600	5.28%	8	0.07%	5.13%
basal forebrain regions	8512	n.d.	n.d.	n.d.	n.d.	84	0.99%	n.d.	n.d.	0.99%
septal regions	1430	n.d.	n.d.	n.d.	n.d.	6	0.42%	n.d.	n.d.	0.42%
thalamus	10693	1	0.01%	1149	**10.75%**	1001	9.36%	2	0.02%	-1.39%
thalamus (stria medullaris)	73	n.d.	n.d.	1	1.37%	3	4.11%	n.d.	n.d.	2.74%
bed nucleus of the stria terminalis	262	n.d.	n.d.	n.d.	n.d.	41	**15.65%**	n.d.	n.d.	**15.65%**
hypothalamic periventricular gray	2750	n.d.	n.d.	197	7.16%	n.d.	n.d.	n.d.	n.d.	-7.16%
pretectal regions	1335	n.d.	n.d.	143	**10.71%**	n.d.	n.d.	n.d.	n.d.	**-10.71%**
superior colliculus (deep layers)	4102	n.d.	n.d.	770	**18.77%**	n.d.	n.d.	n.d.	n.d.	**-18.77%**
inferior colliculus	4624	n.d.	n.d.	256	5.54%	n.d.	n.d.	n.d.	n.d.	-5.54%
substantia nigra	678	n.d.	n.d.	1	0.15%	n.d.	n.d.	n.d.	n.d.	-0.15%
periaqueductal gray	2919	n.d.	n.d.	84	2.88%	n.d.	n.d.	n.d.	n.d.	-2.88%
cerebellum (molecular layer)	17769	n.d.	n.d.	772	4.34%	n.d.	n.d.	n.d.	n.d.	-4.34%
cerebellum (granule cell level)	21465	n.d.	n.d.	1206	5.62%	n.d.	n.d.	n.d.	n.d.	-5.62%
brainstem	31048	n.d.	n.d.	958	3.09%	n.d.	n.d.	n.d.	n.d.	-3.09%
**White Matter**										
corpus callosum*	8733	57	0.65%	n.d.	n.d.	n.d.	n.d.	375	4.29%	-3.64%
anterior commissure	100	n.d.	n.d.	n.d.	n.d.	6	6.00%	n.d.	n.d.	6.00%
anterior commissure, anterior part	366	n.d.	n.d.	n.d.	n.d.	30	8.20%	n.d.	n.d.	8.20%
anterior commissure, posterior part	36	n.d.	n.d.	n.d.	n.d.	1	2.78%	n.d.	n.d.	2.78%
hippocampal fimbria	1417	147	**10.37%**	58	4.09%	12	0.85%	137	9.67%	-2.54%
corticofugal pathways (descending)	3694	n.d.	n.d.	8	0.22%	n.d.	n.d.	n.d.	n.d.	-0.22%
inferior colliculus commissure	170	n.d.	n.d.	44	**25.88%**	n.d.	n.d.	n.d.	n.d.	**-25.88%**
facial nerve (genu)	32	n.d.	n.d.	20	**62.50%**	n.d.	n.d.	n.d.	n.d.	**-62.50%**
**Ventricular System**	3045	12	0.39%	n.d.	n.d.	4	0.13%	32	1.05%	-0.53%

n.d. = not detected

*and associated subcortical white matter
