# Supplementary material for: Jacobian Maps Reveal Under-reported Brain Regions Sensitive to Extreme Binge Ethanol Intoxication in the Rat
Source: Front Neuroanat. 2018 Dec 11;12:108. doi: 10.3389/fnana.2018.00108 (PMC6297262; doi:10.3389/fnana.2018.00108)
Supplement: Supplementary file 2 [file Data_Sheet_2.PDF]

Supplementary Table 2. Within EtOH group analysis using WHS SD atlas

ROI Name	ROI vol	time 1 - 2 expand vol	time 1 - 2 expand %	time 1 - 2 shrink vol	time 1 - 2 shrink %	time 2 - 3 expand vol	time 2 - 3 expand %	time 2 - 3 shrink vol	time 2 - 3 shrink %	Overall
**Gray Matter**										
accessory olfactory bulb (glomerular layer)	151	44	**29.14%**	n.d.	n.d.	2	1.32%	n.d.	n.d.	**30.46%**
olfactory bulb (glomerular layer)	1359	43	3.16%	n.d.	n.d.	56	4.12%	n.d.	n.d.	7.28%
olfactory bulb	15900	852	5.36%	7	0.04%	768	4.83%	n.d.	n.d.	**10.14%**
neocortex	66108	2616	3.96%	3171	4.80%	2310	3.49%	341	0.52%	2.65%
cingulate cortex, area 2	1004	n.d.	n.d.	1	0.10%	n.d.	n.d.	n.d.	n.d.	-0.10%
entorhinal cortex	2241	n.d.	n.d.	208	9.28%	n.d.	n.d.	n.d.	n.d.	-9.28%
lateral entorhinal cortex	2631	n.d.	n.d.	304	**11.55%**	n.d.	n.d.	13	0.49%	**-11.55%**
perirhinal area 35	594	3	0.51%	23	3.87%	n.d.	n.d.	18	3.03%	-3.86%
perirhinal area 36	912	6	0.66%	9	0.99%	n.d.	n.d.	45	4.93%	-3.36%
postrhinal cortex	1214	n.d.	n.d.	133	**10.96%**	n.d.	n.d.	31	2.55%	**-15.89%**
hippocampal formation		n.d.	n.d.	n.d.	n.d.	n.d.	n.d.	n.d.	n.d.	-2.55%
hippocampal cornu ammonis 1	4382	339	7.74%	137	3.13%	737	**16.82%**	355	8.10%	**21.43%**
hippocampal cornu ammonis 2	382	189	**49.48%**	n.d.	n.d.	1	0.26%	168	**43.98%**	**41.64%**
hippocampal cornu ammonis 3	3705	1335	**36.03%**	n.d.	n.d.	340	9.18%	1154	**31.15%**	1.23%
hippocampal dentate gyrus	4570	68	1.49%	n.d.	n.d.	807	**17.66%**	108	2.36%	**-12.00%**
hippocampal fasciola cinereum	479	7	1.46%	n.d.	n.d.	28	5.85%	10	2.09%	4.94%
hippocampal parasubiculum	775	n.d.	n.d.	20	2.58%	n.d.	n.d.	n.d.	n.d.	-4.67%
hippocampal presubiculum	1293	n.d.	n.d.	14	1.08%	n.d.	n.d.	n.d.	n.d.	-1.08%
hippocampal subiculum	2273	n.d.	n.d.	199	8.75%	53	2.33%	n.d.	n.d.	-6.42%
striatum	11354	111	0.98%	335	2.95%	472	4.16%	21	0.18%	2.18%
basal forebrain regions	8512	n.d.	n.d.	475	5.58%	375	4.41%	n.d.	n.d.	-1.36%
subthalamic nucleus	17	n.d.	n.d.	n.d.	n.d.	8	**47.06%**	n.d.	n.d.	**47.06%**
septal regions	1430	250	**17.48%**	76	5.31%	16	1.12%	n.d.	n.d.	**13.29%**
thalamus	10693	46	0.43%	406	3.80%	1408	**13.17%**	n.d.	n.d.	9.80%
thalamus (stria medullaris)	73	n.d.	n.d.	n.d.	n.d.	8	**10.96%**	n.d.	n.d.	**10.96%**
bed nucleus of the stria terminalis	262	n.d.	n.d.	5	1.91%	32	**12.21%**	n.d.	n.d.	**10.31%**
hypothalamic regions	2702	n.d.	n.d.	156	5.77%	589	**21.80%**	n.d.	n.d.	**16.03%**
hypothalamic periventricular gray	2750	n.d.	n.d.	300	**10.91%**	n.d.	n.d.	n.d.	n.d.	**-10.91%**
pretectal regions	1335	n.d.	n.d.	229	**17.15%**	55	4.12%	n.d.	n.d.	**-13.03%**
superior colliculus (superficial gray)	863	n.d.	n.d.	n.d.	n.d.	1	0.12%	n.d.	n.d.	0.12%
superior colliculus (deep layers)	4102	n.d.	n.d.	675	**16.46%**	202	4.92%	n.d.	n.d.	**-11.54%**
inferior colliculus	4624	n.d.	n.d.	361	7.81%	n.d.	n.d.	n.d.	n.d.	**-7.81%**
substantia nigra	678	383	**56.49%**	17	2.51%	96	**14.16%**	n.d.	n.d.	**68.14%**
interpeduncular nucleus	141	114	**80.85%**	n.d.	n.d.	n.d.	n.d.	n.d.	n.d.	**80.85%**
periaqueductal gray	2919	n.d.	n.d.	37	1.27%	6	0.21%	n.d.	n.d.	-1.06%
pontine nuclei	497	75	**15.09%**	n.d.	n.d.	n.d.	n.d.	n.d.	n.d.	**15.09%**
cerebellum (molecular layer)	17769	749	4.22%	47	0.26%	n.d.	n.d.	n.d.	n.d.	3.95%
cerebellum (granule cell level)	21465	1034	4.82%	331	1.54%	n.d.	n.d.	n.d.	n.d.	3.28%
facial nerve (genu)	32	n.d.	n.d.	7	**21.88%**	n.d.	n.d.	n.d.	n.d.	**-21.88%**
spinal trigeminal nuclus	1810	n.d.	n.d.	61	3.37%	n.d.	n.d.	n.d.	n.d.	-3.37%
spinal trigeminal tract	6664	25	0.38%	41	0.62%	86	1.29%	n.d.	n.d.	1.05%
brainstem	31048	1143	3.68%	1711	5.51%	156	0.50%	n.d.	n.d.	-1.33%
**White Matter**										
corpus callosum	8733	157	1.80%	855	9.79%	4	0.05%	316	3.62%	-7.95%
anterior commissure	100	n.d.	n.d.	1	1.00%	17	**17.00%**	n.d.	n.d.	**12.38%**
anterior commissure, anterior part	366	n.d.	n.d.	170	**46.45%**	31	8.47%	n.d.	n.d.	**-37.98%**
anterior commissure, posterior part	36	n.d.	n.d.	4	**11.11%**	6	**16.67%**	n.d.	n.d.	5.56%
hippocampal alveus	10	6	**60.00%**	n.d.		n.d.	n.d.	n.d.	n.d.	**60.00%**
hippocampal (ventral) commissure	155	n.d.	n.d.	17	**10.97%**	n.d.	n.d.	n.d.	n.d.	**-10.97%**
fornix	135	n.d.	n.d.	21	**15.56%**	n.d.	n.d.	n.d.	n.d.	**-15.56%**
mammillothalamic tract	6	n.d.	n.d.	1	**16.67%**	n.d.	n.d.	n.d.	n.d.	**-16.67%**
hippocampal fimbria	1417	304	**21.45%**	7	0.49%	59	4.16%	205	**14.47%**	**25.12%**
corticofugal pathways (descending)	3694	263	7.12%	38	1.03%	60	1.62%	n.d.	n.d.	-6.75%
medial lemniscus	85	3	3.53%	34	**40.00%**	2	2.35%	n.d.	n.d.	**-34.12%**
optic tract and optic chiasm	345	n.d.	n.d.	n.d.	n.d.	3	0.87%	n.d.	n.d.	0.87%
inferior colliculus commissure	170	n.d.	n.d.	19	**11.18%**	n.d.	n.d.	n.d.	n.d.	**-11.18%**
inferior cerebellar peduncle	565	53	9.38%	21	3.72%	n.d.	n.d.	n.d.	n.d.	5.66%
middle cerebellar peduncle	304	n.d.	n.d.	1	0.33%	n.d.	n.d.	n.d.	n.d.	-0.33%
pons transverse fibers	261	61	**23.37%**	n.d.	n.d.	n.d.	n.d.	n.d.	n.d.	**23.37%**
**Ventricular System**	3045	113	3.71%	6	0.20%	32	1.05%	53	1.74%	4.56%

n.d. = not detected

*and associated subcortical white matter
