# Supplementary material for: Jacobian Maps Reveal Under-reported Brain Regions Sensitive to Extreme Binge Ethanol Intoxication in the Rat
Source: Front Neuroanat. 2018 Dec 11;12:108. doi: 10.3389/fnana.2018.00108 (PMC6297262; doi:10.3389/fnana.2018.00108)
Supplement: Supplementary file 3 [file Data_Sheet_3.PDF]

Supplementary Table 3. Within control group analysis using WHS SD atlas

ROI Name	ROI Volume	time 1 - 2				time 2 - 3				Overall
		expand vol	expand %	shrink vol	shrink %	expand vol	expand %	shrink vol	shrink %	
**Gray Matter**										
neocortex	66108	n.d.	n.d.	n.d.	n.d.	n.d.	n.d.	1107	1.67%	-1.67%
frontal associiation cortex	1019	n.d.	n.d.	n.d.	n.d.	n.d.	n.d.	17	1.67%	-1.67%
entorhinal cortex	2241	n.d.	n.d.	n.d.	n.d.	n.d.	n.d.	43	1.92%	-1.92%
lateral entorhinal cortex	2631	n.d.	n.d.	n.d.	n.d.	n.d.	n.d.	343	**13.04%**	**-13.04%**
hippocampal formation		n.d.	n.d.	n.d.	n.d.	n.d.	n.d.	n.d.	n.d.	
hippocampal cornu ammonis 1	4382	n.d.	n.d.	n.d.	n.d.	27	0.62%	n.d.	n.d.	0.62%
hippocampal cornu ammonis 3	3705	n.d.	n.d.	n.d.	n.d.	65	1.75%	n.d.	n.d.	1.75%
hippocampal dentate gyrus	4570	n.d.	n.d.	n.d.	n.d.	259	5.67%	n.d.	n.d.	5.67%
hippocampal fasciola cinereum	479	n.d.	n.d.	n.d.	n.d.	31	6.47%	n.d.	n.d.	6.47%
hippocampal presubiculum	1293	n.d.	n.d.	n.d.	n.d.	n.d.	n.d.	256	**19.80%**	**-19.80%**
hippocampal subiculum	2273	n.d.	n.d.	n.d.	n.d.	n.d.	n.d.	54	2.38%	-2.38%
striatum	11354	n.d.	n.d.	n.d.	n.d.	1	0.01%	n.d.	n.d.	0.01%
basal forebrain regions	8512	n.d.	n.d.	n.d.	n.d.	n.d.		34	0.40%	-0.40%
thalamus	10693	n.d.	n.d.	n.d.	n.d.	905	8.46%	4	0.04%	8.42%
thalamus (stria medullaris)	73	n.d.	n.d.	n.d.	n.d.	1	1.37%	n.d.	n.d.	1.37%
stria terminalis	121	n.d.	n.d.	n.d.	n.d.	2	1.65%	n.d.	n.d.	1.65%
hypothalamic regions	2702	n.d.	n.d.	n.d.	n.d.	n.d.	n.d.	9	0.33%	-0.33%
hypothalamic periventricular gray	2750	5	0.18%	n.d.	n.d.	n.d.	n.d.	n.d.	n.d.	0.18%
pretectal regions	1335	n.d.	n.d.	n.d.	n.d.	111	8.31%	n.d.	n.d.	8.31%
superior colliculus (superficial gray)	863	n.d.	n.d.	n.d.	n.d.	n.d.	n.d.	72	8.34%	-8.34%
superior colliculus (deep layers)	4102	n.d.	n.d.	n.d.	n.d.	2	0.05%	44	1.07%	-1.02%
inferior colliculus	4624	n.d.	n.d.	n.d.	n.d.	1	0.02%	84	1.82%	-1.79%
cerebellum (molecular layer)	17769	487	2.40%	n.d.	n.d.	n.d.	n.d.	167	0.94%	1.46%
cerebellum (granule cell level)	21465	516	2.74%	n.d.	n.d.	n.d.	n.d.	73	0.34%	2.40%
brainstem	31048	14	0.05%	n.d.	n.d.	1	0.00%	181	0.58%	-0.53%
**White Matter**										
hippocampal fimbria	1417	n.d.	n.d.	n.d.	n.d.	96	6.77%	n.d.	n.d.	6.77%
corticofugal pathways (descending)	3694	n.d.	n.d.	n.d.	n.d.	2	0.05%	1	0.03%	0.03%
optic tract and optic chiasm	345	n.d.	n.d.	n.d.	n.d.	n.d.	n.d.	21	6.09%	-6.09%
spinal trigeminal tract	6664	n.d.	n.d.	n.d.	n.d.	n.d.	n.d.	230	3.45%	-3.45%
middle cerebellar peduncle	304	n.d.	n.d.	n.d.	n.d.	n.d.	n.d.	43	**14.14%**	**-14.14%**
**Ventricular System**	3045	n.d.	n.d.	n.d.	n.d.	19	0.62%	112	3.68%	-3.05%

n.d. = not detected
